# Engineering Stable Lentiviral Vector Producer Cells Improves LVV Production

**DOI:** 10.1002/bit.70318

**Published:** 2026-07-21

**Authors:** Jona Röscheise, Lena‐Marie Eberle, Holger Laux, Kerstin Otte

**Affiliations:** ^1^ Institute of Applied Biotechnology Biberach University of Applied Sciences Biberach Germany; ^2^ Department of Gene Therapy University Ulm Ulm Germany; ^3^ Biopharmaceutical Product Development CSL Behring Innovation GmbH Marburg Germany

## Abstract

Stable lentiviral vector producer cell lines represent a promising platform for scalable and cost‐efficient vector manufacturing, yet their productivity is often limited by intrinsic host‐cell constraints. In this study, we aimed to identify cellular factors restricting LVV production in stable doxycycline‐inducible GPRTG producer cell lines and to evaluate whether targeted host‐cell engineering can improve vector yield. Comparative transcriptomic analysis of high‐ and low‐producing clones revealed distinct differences in cellular pathways related to transcription, translation, energy metabolism, lipid homeostasis, stress response, and chromatin regulation. Based on differential gene expression, candidate genes were modified by CRISPR/Cas9‐mediated knockout or overexpression and functionally analyzed in a low‐producing clone. Single‐gene KO screening identified H1‐2, ADAMTS1, INSIG1, GADD45B, and HSPA1B to increase cell‐specific LVV productivity up to 2.6‐fold, with H1‐2 showing the strongest effect. In addition, combinatorial disruption further improved productivity. In contrast, overexpression of selected candidates did not enhance LVV production. Overall, our results demonstrate that transcriptomics‐guided host‐cell engineering is an effective strategy to identify and relieve intrinsic bottlenecks in stable LVV producer cell lines.

**Abbreviations**
Cas9: CRISPR‐associated protein 9
cDNA: complementary DNA
CPM: counts per millions
CRISPR: clustered regularly interspaced short palindromic repeats
Dox: doxycycline
FC: fold change
Gag: group‐specific antigen
gDNA: genomic DNA
H: hours
ICE: interference of CRISPR edits
InDel: insertions or deletions
KO: knockout
LDLR: low‐density lipoprotein receptor
LVVs: lentiviral vectors
Min: minutes
OEX: overexpression
PCA: principal component analysis
POL: polymerase
qPCR: quantitative PCR
Rev: regulator of expression of virion proteins
RF: restriction factor
RNA: ribonucleic acid
RNP: ribonucleoprotein
Rpm: rounds per minute
sgRNA: single‐guide RNA
SIN‐MLV: self‐inactivating gamma‐retroviral
Tat: trans‐activator of transcription
TPM: transcripts per millions
VCD: viable cell density
VSV‐G: vesicular stomatitis virus glycoprotein G

## 1 Introduction

Lentiviral vectors (LVVs) are a valuable tool for gene therapy applications, due to the possibility to integrate a transgene of interest stably into both dividing and non‐dividing cells (Throm 2009). Traditional LVV production is performed through co‐transfection of plasmids carrying the necessary sequences for LVV replication (Durand and Cimarelli 2011). However, this approach needs large amounts of purified DNA, is prone to batch‐to‐batch variabilities, and requires a lot of transfection reagent, which results in a cost‐intensive production process. The creation of stable producer cell lines with the genes integrated necessary for LVV replication enables a more consistent, scalable, and cost‐effective alternative to the traditional approach (Broussau 2023; Ferreira et al. 2021).

Despite their clear advantages, stable producer cell lines can still show limited productivity due to cellular defense mechanisms or self‐transduction of newly formed LVV particles. Recent research revealed several host factors, also called restriction factors (RF), that target necessary steps in LVV replication and inhibit the life cycle (Kajaste‐Rudnitski 2009; Kajaste‐Rudnitski and Naldini 2015; Kmiec and Kirchhoff 2024; Strebel et al. 2009). Some of those RFs are counterattacked by viral proteins. APOBEC3 proteins restrict HIV‐1 by deaminating viral complementary DNA (cDNA) during reverse transcription; this effect is neutralized by the viral protein Vif, which promotes APOBEC3 degradation (Kmiec and Kirchhoff 2024). Similarly, Tetherin (BST2) prevents virion release by retaining particles at the cell surface, a restriction that is counteracted by HIV‐1 Vpu through downregulation of Tetherin (Kmiec and Kirchhoff 2024).

RFs are not limited to classical antiviral proteins but also include host genes that influence different stages of the lentiviral life cycle. For LVV production, the most widely used envelope glycoprotein for pseudo‐typing is the vesicular stomatitis virus glycoprotein G (VSV‐G), owing to its high stability and broad tropism. Entry of VSV‐G–pseudotyped LVVs is primarily mediated by the low‐density lipoprotein receptor (LDLR), although other members of the LDLR family can function as alternative entry receptors. If the LDLR receptor is present on the production cell line, viral entry by self‐transduction can be mediated, and a loss of LVV particles occurs. The research team among Han showed that a knockout (KO) of LDLR in HEK293 LVV producing cells can increase LVV titer by 2.0‐fold (Han et al. 2021).

Overall, the spectrum of host RFs is not yet fully defined, and numerous cellular genes may restrict pathways that support efficient LVV assembly and release without being directly involved in the viral replication machinery. Such processes may include lipid biosynthesis required for membrane formation, regulation of transcriptional and translational capacity, intracellular trafficking, and cellular stress or protein quality control pathways. Supporting this concept, Zhang et al. performed a Clustered Regularly Interspaced Short Palindromic Repeats/CRISPR‐associated protein 9 (CRISPR/Cas9)‐based high‐throughput screen and identified nine genes, out of 17,501 analyzed, that significantly influenced LVV replication (Xinyue 2024). These candidate genes were associated with diverse cellular functions, including innate immunity (GBP3), phospholipid binding (BPIFC), transcriptional repression (ZNF425), RNA degradation (TTC47), clearance of toxic protein aggregates (NHLRC1), and lipid metabolism (LDAH) (Xinyue 2024). Notably, KO of LDAH in HEK293T producer cells resulted in a marked increase in viral titers (Xinyue 2024).

In 2009, Throm et al. developed stable lentiviral packaging and producer cell lines to enable consistent and scalable vector manufacturing (Throm et al. 2009). The GPRTG system was established from HEK293T/17 cells by stepwise integration of lentiviral packaging components using self‐inactivating gamma‐retroviral (SIN‐MLV) vectors. In these cells, group‐specific antigen (Gag) and polymerase (Pol) are constitutively expressed, whereas regulator of expression of virion proteins (Rev), trans‐activator of transcription (Tat), and the vesicular stomatitis virus glycoprotein (VSV‐G) envelope protein are controlled by a tetracycline‐inducible promoter, allowing regulated vector production while maintaining biosafety (Throm et al. 2009). Although this producer line provides a robust platform for LVV generation, substantial differences in productivity between individual clones suggest that intrinsic host‐cell factors may limit vector yield.

In this work, we therefore aimed to systematically identify cellular constraints within this specific production system and to define molecular determinants that restrict LVV output. To identify host cell determinants limiting LVV productivity, we performed a comparative transcriptomic analysis of high‐ and low‐producing cell lines. This unbiased approach enabled the identification of genes and pathways associated with productive versus suboptimal vector output. Genes enriched in low‐producing cells were considered potential inhibitory factors, while genes overrepresented in high‐producing cells were identified as candidates for targeted enhancement. Beyond classical antiviral responses, this strategy captured broader cellular processes such as metabolism, membrane biogenesis, protein processing, and stress responses.

## 2 Material and Methods

### 2.1 Cell Culture, Induction and RNA Extraction

The generation of the used GPRTG cell lines was described in 2009. Although the integrated viral expression cassettes were generated using related vector designs, expression of the integrated components may still be influenced by the genomic integration site, local chromatin environment, and clonal variation. Therefore, productivity differences between clones cannot be attributed solely to vector‐backbone features. Suspension GPRTG cells were cultured in HyCell TransFx‐H media (Cytiva) supplemented with 6 mM GlutaMAX (Thermo Fisher Scientific, Schwerte, Germany), 1.9 g/L Cell Boost 5 stock solution (Cytiva, Marlborough, MA, US), 0.1% (w/v) Pluronic F‐68 Non‐ionic Surfactant (Thermo Fisher Scientific), and 0.01% (v/v) anti‐clumping agent (Thermo Fisher Scientific). Cell Boost 5 was identified through a media screening according to Klimpel et al. (2023). Pluronic functions as a shear stress protectant, the anti‐clumping agent prevents aggregate formation and glutamine is an essential component in cell culture media. The medium was supplemented at each passage with 2.5 ng/mL doxycycline (Dox) for suspension cultures. The cell culture was cultivated in a 37°C shaking incubator at 120 revolutions per minute (rpm) (25 mm orbit), 5% CO2 and 85% humidity. GPRTG cells were passaged every 3–4 days. Viability and viable cell density (VCD) were monitored using Nucleocounter NC200 with Via1‐Cassettes (ChemoMetec A/S, Allerod, Denmark) following the manufacturer's instructions.

For LVV production, cells were seeded at 8 * 10^5 ^cells/mL in a 125 mL shaking flask and cultivated −Dox in batch‐mode for 72 h at 37°C.

After 72 h, cultures were transitioned to semi‐perfusion mode and maintained for an additional 6 days. Daily, cells were centrifuged (800 rpm, 2 min) and LVV‐containing supernatant was collected.

RNA was isolated from 3 * 10^6^ cells harvested on day six using RNeasy plus mini kit (QIAGEN GmbH, Düsseldorf, Germany) according to the manufacturer's protocol. For lysis, 1 mL lysis buffer was supplemented with freshly prepared 20 µL 2 M DTT solution. 2 M DTT solution (Sigma Aldrich, Taufkirchen, Germany) with MilliQ‐H_2_O.

For both cultivation conditions (−Dox and +Dox), three biological replicates were analyzed.

### 2.2 Library Preparation and Sequencing

RNA quality control and cDNA synthesis were performed by Eurofins (Ebersberg, Germany).

All samples showed a 260/280 ratio of 1.9–2.1, a 260/230 ratio of 2.0–2.2, and an RNA integrity number (RIN) > 8. cDNA libraries were generated using the NEBNext Ultra II Directional RNA Library Prep Kit for Illumina (New England Biolabs GmbH, Frankfurt). Adapter ligation and PCR amplification were performed according to the manufacturer's protocol to enrich for properly ligated fragments. (Illumina, San Diego, CA, US).

### 2.3 Sequence Alignment and Gene Counts

All high‐quality sequenced reads were aligned to the human reference genome (GRCh38) supplemented with custom sequences encoding the integrated viral transgenes (STAR, Version 2.7.8a).

### 2.4 Differential Gene Expression

Low‐expressed genes with CPM values less than 1 were removed prior to downstream analysis. Genes with CPM values higher 1 were analyzed. Normalization was performed using calcNormFactors (edgeR), which estimates scaling factors to minimize log fold changes (FCs) between samples.

Statistical tests (exact test of Robinson and Smyth (2007)) applied to compare expression distributions between conditions. Resulting *p* values were adjusted for false discovery rate (FDR) using the Benjamini–Hochberg method.

### 2.5 Selection of Candidate Genes for KO and OEX Using Venn Analysis

Differential gene expression analyses were used to identify candidate genes for KO or overexpression (OEX) based on comparisons between high‐producing (clones 40 and 42) and low‐producing (clones 29 and 33) clones. For KO target identification, genes were filtered by a transcripts per million (TPM) value of ≥ 10 in low producers, no TPM threshold in high producers, and a Log_2_ fold change (Log_2_FC) ≤ −0.6 (corresponding to a non‐log FC ≤ 1.5).

In contrast, OEX targets were defined by a TPM of ≥ 10 in high producers, no TPM cutoff in low producers, and a Log_2_FC ≥ 0.6.

Filtering was performed separately for both induced (−Dox) and non‐induced (+Dox) conditions. Gene sets were compared using Venn diagrams to identify condition‐specific and shared candidates. Genes uniquely present in the −Dox condition, as well as those shared between both conditions, were prioritized for further evaluation.

Selected candidates were further examined based on functional annotation, relevance to viral replication, and their potential impact on viral production. Final ranking of candidate genes was guided by Log_2_FC magnitude, expression levels, and literature‐based associations with viral biology.

### 2.6 Single Guide RNA and OEX Construct Generation

Gene KO was achieved using the CRISPR/Cas9 system by introducing small insertions or deletions (InDels) through a single‐guide RNA (sgRNA)‐directed Cas9 nuclease. For gene OEX, full‐length coding sequences of selected targets were amplified from cDNA generated from total RNA isolated from HEK293T/17 cells, as described in Section 2.9. PCR amplification was performed using gene‐specific primers containing BamHI and NotI restriction overhangs, and the resulting amplicons were cloned into a pcDNA3.1‐based mammalian expression vector under control of the CMV promoter. The amplified target gene sequences and corresponding forward and reverse primers are listed in Table 1. Of the 13 candidate genes initially selected for OEX, three constructs (DBI, ID3, and TIMM8B) were successfully cloned and used for subsequent transient overexpression experiments.

**Table 1:** Primers used for amplification of coding sequences for OEX constructs. The table lists the target gene, NCBI RefSeq accession number used as coding‐sequence reference, and the corresponding forward and reverse primers used for amplification and cloning.

Target gene	Gene accession	Forward primer	Reverse primer
ID3	NM_002167.5	ATATATGGATCCATGAAG GCGCTGAGCCC	ATATATGCGGCCGCTCAGTGGCA AAAGCTCCTTTTG
DBI	NM_020548.9	ATATATGGATCCTATGTG GGGCGACCTCTG	ATATATGCGGCCGCTCATATCCC GTATTTTTTCTTTAGCTCTT
Timm8B	NM_012459.4	ATATGGATCCATGGCGGA GCTGGGCG	ATATGCGGCCGCCTACTGCCCT CCTTTCTGTACAATCTG

### 2.7 sgRNA and OEX Construct Transfection

For KO experiments, 1250 ng of recombinant Cas9 protein (Thermo Fisher Scientific) was complexed with 7.5 pmol sgRNA (Integrated DNA Technologies or Merck) and incubated at room temperature for 5–20 min to allow ribonucleoprotein (RNP) complex formation.

All transfections were carried out in the low‐producing clone 33 (cl33) using the Neon Transfection System (Thermo Fisher Scientific). The 10 µL tip format was used for RNP delivery, and the 100 µL tip format was used for plasmid‐based OEX. For the 10 µL format, 1 * 10^6^ cells were pelleted by centrifugation, resuspended in R buffer containing the pre‐formed RNP complex, and electroporated using the following settings: 1100 V, 20 ms pulse width, and two pulses. For the 100 µL format, 2 * 10^6^ cells were processed similarly, using R buffer containing 4 µg of OEX plasmid, and electroporated with identical electrical parameters. For OEX experiments, clone 33 cells were transiently transfected with OEX plasmids. Transfected cells were not antibiotic‐selected, and no stable OEX cell lines were generated. As a control, cells transfected with an empty vector were used.

### 2.8 Cutting Efficiency Assessment

To evaluate the cutting efficiency of each sgRNA, cells were transfected and harvested 72 h post‐transfection. A total of 1–2*10^5^ cells were collected for genomic DNA (gDNA) extraction using quick extract gDNA buffer (Biozym). The targeted genomic region flanking the CRISPR/Cas9 cleavage site was amplified by PCR using site‐specific primers and Genaxxon polymerase (Genaxxon Bioscience). PCR products were visualized via agarose gel electrophoresis, and bands corresponding to the expected size were excised and purified using a gel extraction kit (Macherey‐Nagel).

Purified PCR products were submitted for Sanger sequencing (Eurofins Genomics), and resulting electropherograms were analyzed for editing efficiency using the Synthego Inference of CRISPR Edits (ICE) analysis platform. ICE deconvolutes mixed Sanger sequencing traces by comparison to a wild‐type control to infer the frequency and distribution of CRISPR‐induced indels, reporting the overall editing efficiency (ICE score) and indel spectrum at the target site.

### 2.9 RNA Extraction and Gene Expression Analysis Using Quantitative PCR

Total RNA was extracted from 1 * 10^6^ cells using QIAzol Lysis Reagent (Qiagen) following the manufacturer's instructions. RNA was eluted in 30 µL RNase‐free water and quantified using a Nanodrop spectrophotometer.

For cDNA synthesis, 1 µg of total RNA was reverse transcribed in a 20 µL reaction using the High‐Capacity cDNA Reverse Transcription Kit (Thermo Fisher Scientific) with random primers, dNTPs, MultiScribe Reverse Transcriptase, and RNase Inhibitor. Reactions were incubated in a thermal cycler under the following conditions (Table 2).

**Table 2:** Settings for reverse transcription.

Settings	Step 1	Step 2	Step 3	Step 4
Temperature	25°C	37°C	85°C	4°C
Time	10 min	120 min	5 min	hold

The resulting cDNA was diluted 1:20 with RNase‐free water for quantitative PCR (qPCR) amplification using 2× SYBR green PCR mix according to manufacturer's protocol (Genaxxon). qPCR amplification was performed using LightCycler 480 (Roche Diagnostics) with the following settings (Table 3).

**Table 3:** Settings for PCR amplification.

	Temperature (°C)	Time (min)	Ramp rate (°C(s))	Cycles
Pre‐incubation	95	900	4.4	1x
Amplification	95	20	2.2	45x
	57‐62	30	2.2	
	72	20	4.4	
Melting	95	10	4.4	1x
	50	30	2.2	
Cooling	40	30	2.2	1x

### 2.10 Infectious Titer Analysis

To quantify functional LVVs present in cell culture supernatants, a transduction‐based infectivity assay was conducted using adherent HEK293T/17 cells. Cells were maintained under standard culture conditions at 37°C and 5% CO_2_ in DMEM (Thermo Fisher Scientific) supplemented with 10% fetal bovine serum (FBS) and 1% Penicillin‐Streptomycin (Thermo Fisher Scientific). The assay was performed in 96‐well plate formats.

In the 96‐well format, cells were plated at 2 * 10^4^ cells per well in 80 µL of transduction medium. Test samples were diluted in a four‐step series (1:50, 1:250, 1:1250, and 1:6250). The tracking control was diluted 1:50,000. A volume of 20 µL of each diluted sample or control was added to the 80 µL seeded cells, followed by mixing and incubation for 4 days under the same culture conditions.

After 96 h, cells were washed with phosphate buffered saline (PBS), detached using TrypLE Express (Thermo Fisher Scientific) for 3 min at 37°C, and neutralized with DMEM containing FBS. Fixation was performed either using the BD Biosciences Fixation and Permeabilization Kit, following the manufacturer's protocol, or with 4% paraformaldehyde (PFA), followed by two PBS washes. Fixed cells were resuspended in 100 µL of MACS Running Buffer (Miltenyi Biotec) and analyzed for GFP expression via flow cytometry using a CytoFLEX system (Beckman Coulter Life Sciences).

## 3 Results

### 3.1 Characterization of High and Low LVV Producing Cell Clones

Improving LVV yields by cell engineering approaches requires the systematic identification of host‐cell genes that promote or limit vector productivity. To uncover such modulatory genes, individual monoclonal producer cell lines were derived from previously established stable, dox‐inducible GPRTG production cell lines, with clone numbers referring to distinct monoclonal populations (Figure 1A) (Röscheise et al. 2025). These GFP‐encoding producer clones were expanded under non‐induced conditions to allow unhindered growth, followed by induction of LVV production by withdrawal of dox. Supernatants were collected over six consecutive days and pooled to transduce adherent HEK293T/17 cells, and absolute infectious titers were quantified by flow cytometry 4 days post‐transduction. Selected high‐ and low‐producing clones were subjected to transcriptomic profiling and differential gene expression analysis to identify candidate genes associated with cellular productivity (Figure 1A).

**Figure 1:**
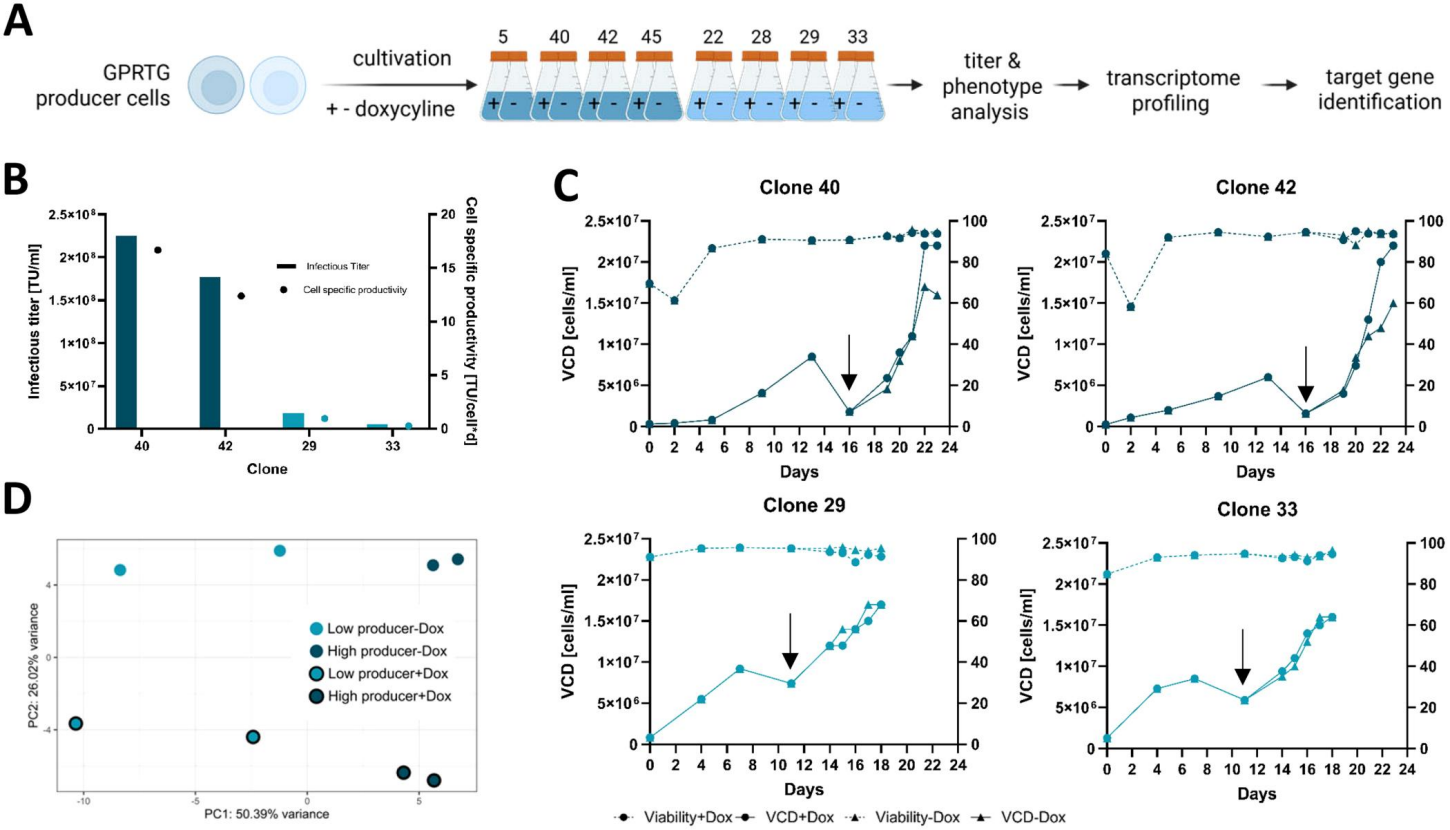
Screening and characterization of stable LVV production clones. (A) Experimental workflow: GPRTG producer cells were cultured, induced by removing doxycycline, and analyzed by titer/phenotype assays, followed by transcriptome profiling and target identification. (B) Antibody titers and specific productivity of selected clones (40, 42, 29, 33). (C) Time‐course of viability, viable cell density (VCD), and productivity; arrows indicate doxycycline removal. (D) PCA plot showing transcriptomic differences between low‐ and high‐producing clones under induced (+Dox) and non‐induced (−Dox) conditions.

Based on generated infectious titer data, two high‐producing clones (40 and 42; 2.25 * 10^8^ and 1.77 * 10^8^ TU/mL, respectively) and two low‐producing clones (29 and 33; 1.83 * 10^7^ and 5.43 * 10^6^ TU/mL, respectively) were selected for further analysis (Figure 1B). Analysis of VCD (Figure 1C, solid lines) during non‐induced cultivation showed increasing cell growth for all clones, reaching on average 6.95 * 10^6^ cells/mL (Figure 1C), whereas induction of high LVV production clones resulted in reduced cell proliferation from day 21 on (Figure 1C, triangle shaped symbols), while maintaining viabilities above 90%.

For transcriptomic analysis, cell pellets were harvested at the end of the production phase (Day 6 after induction) from both non‐induced and induced cultures in biological triplicates. Because individual monoclonal producer lines differed in pre‐culture expansion kinetics, induction was initiated once cultures reached 6 * 10^6^ cells/mL after three passages, the predefined production. Generated RNA sequencing data were subsequently subjected to differential gene expression analysis, comparing high‐ versus low‐producing clones to identify candidate genes associated with LVV titers. Principal component analysis (PCA) of normalized gene expression values revealed clustering of clones into high‐ and low‐producing groups, with less pronounced clustering between presence and absence of dox (Figure 1D). The first two principal components explained ~76% of the total variance (PC1: 50.4%, PC2: 26.0%). High‐ and low‐producing clones separated along PC1, while induction states contributed to additional separation along PC2. The PCA supported the productivity‐based clone classification, as high‐ and low‐ producing clones separated primarily along PC1, whereas the induction state contributed to additional separation along PC2. Thus, the clones selected based on infectious LVV titers also displayed distinct global transcriptomic profiles and were therefore used for downstream differential expression and target‐selection analyses, to identify potential production‐modulating genes for subsequent cell engineering purposes.

### 3.2 Pathway Analysis Highlights Regulatory Differences Between Producer Clones

To identify general pathway‐level differences between high‐ and low‐producing clones, differential gene expression analysis of high (mean of clone 40 and 42) versus low (mean of clone 29 and 33) producers was calculated from transcriptome data under both induced and non‐induced conditions.

Ingenuity Pathway Analysis (IPA) analysis showed that in the non‐induced state, the main pathways that differed between the clones were upregulated in the high‐producing clones than in the low‐producing ones (Figure 2A). These pathways were mainly related to protein production, gene expression, and energy metabolism. In contrast, mitochondrial dysfunction was downregulated, consistent with enhanced metabolic capacity.

**Figure 2:**
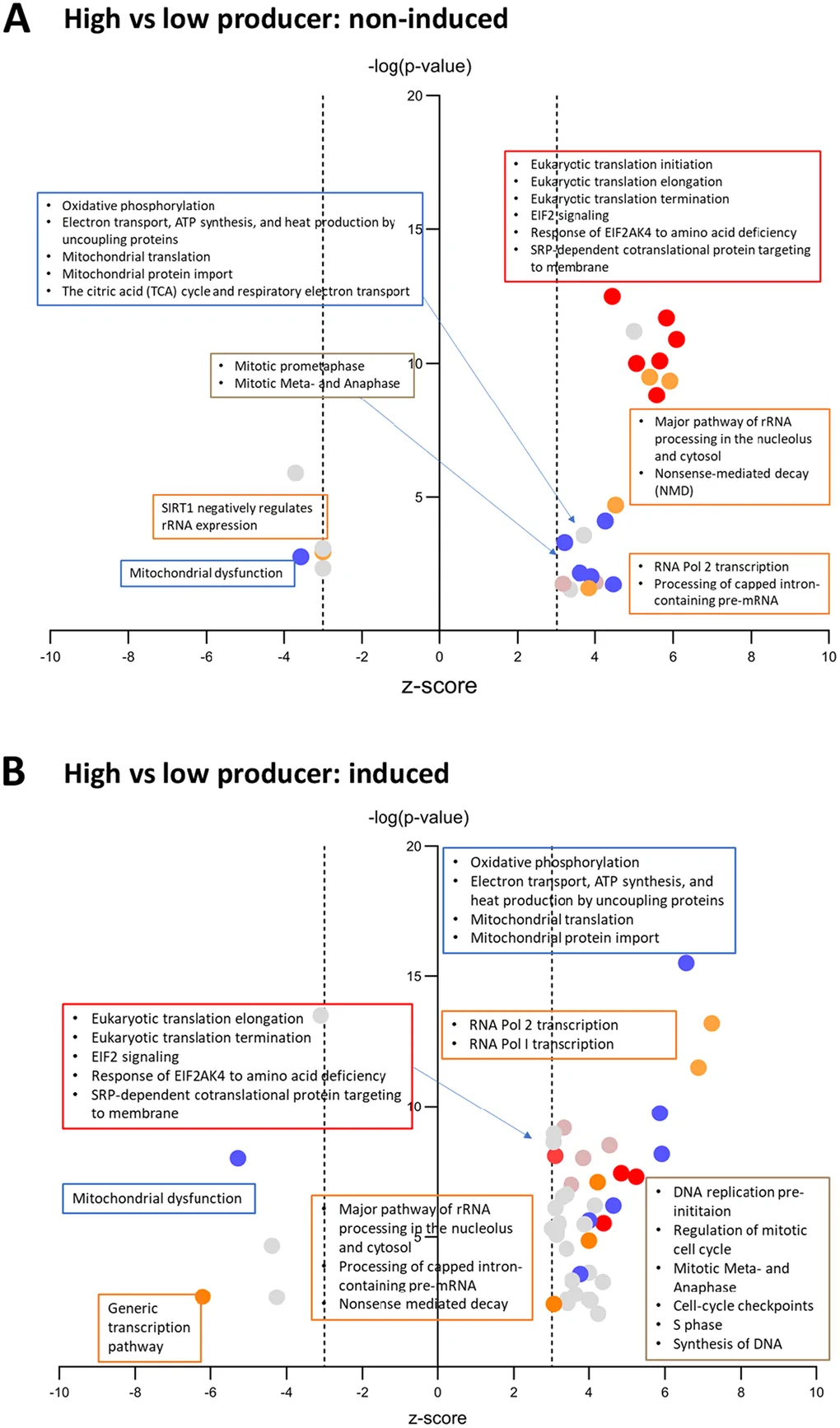
Differential pathway regulation analysis of between high and low producing clones induced and non‐induced. (A/B) Predicted pathway regulation based on the comparison of LVV high production cell lines compared to low production cell lines, non‐induced (A) and induced (B).

Upon induction and full LVV production, pathway regulation largely mirrored the non‐induced state, though with altered strength and significance: translation‐ and transcription‐related pathways remained upregulated (with reduced statistical significance), energy‐related pathways were further enhanced, and mitochondrial dysfunction remained strongly downregulated (Figure 2B). Notably, cell cycle‐related pathways, including DNA synthesis, S phase, and checkpoints, were upregulated in high producers, a pattern absent in the non‐induced comparison (Figure 2A).

Overall, high‐producing clones exhibit enhanced biosynthetic, metabolic, and cell cycle‐related pathway activity compared to low producers, both before and after LVV induction, supporting their superior capacity for viral production.

### 3.3 Gene Expression Analysis Identifies LVV Engineering Target Genes

To initially assess the generated transcriptome datasets, gene expression levels across the different cell clones and culture conditions were analyzed. Most genes (around 9400) were found to be non‐ or lowly expressed (TPM < 10), while around 5000 genes showed medium expression (TPM 10–100), and only a few genes were highly expressed (TPM > 100) (Figure 3A). Overall, the distribution of genes across these TPM classes was consistent between conditions. Given that antiviral RFs may serve as potential engineering targets to enhance LVV production, we compiled a literature‐based list of 395 previously reported RFs and retrieved their expression data from our transcriptome dataset. Most RFs (around 213 genes) were lowly or not expressed (TPM < 10), around 125 genes showed medium expression (TPM 10–100), and around 55 genes were highly expressed (TPM > 100) (Figure 3B). Again, the distribution of genes across these TPM classes was consistent between conditions.

**Figure 3:**
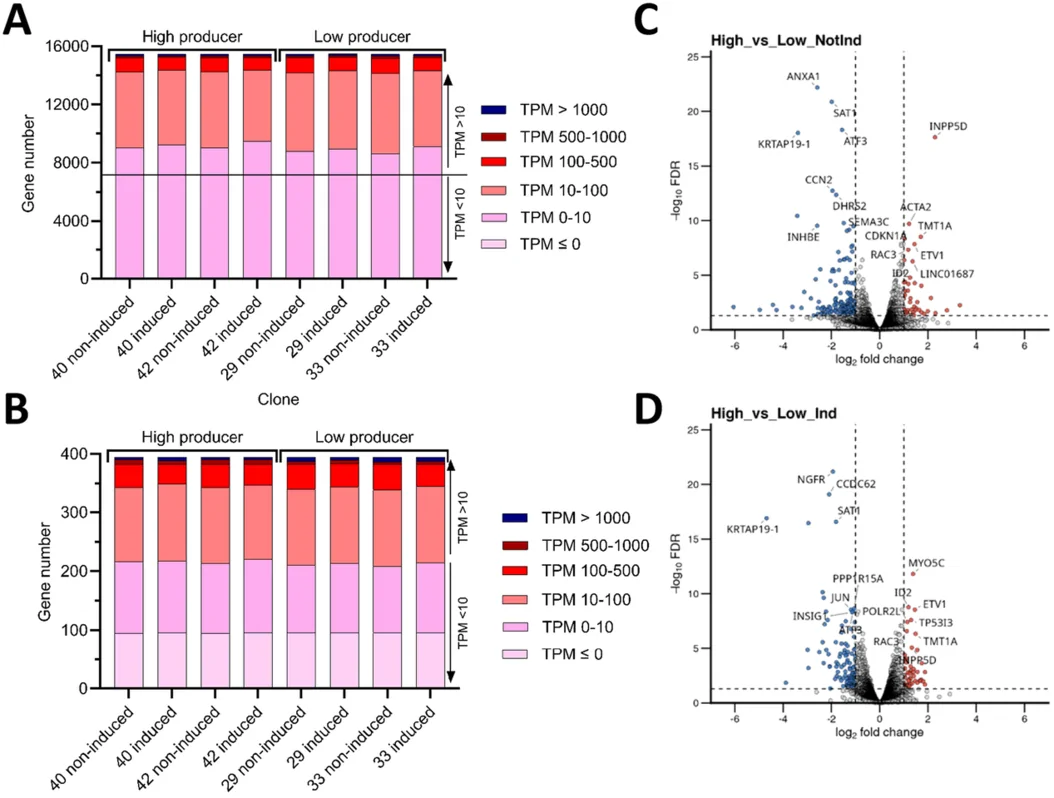
Transcriptomic comparison of high‐ and low‐producing clones. (A–B) Distribution of gene expression levels (TPM) across clones under induced and non‐induced conditions, shown for high and low producers. (C–D) Volcano plots of differential gene expression between high‐ and low‐producing clones under non‐induced (C) and induced (D) conditions; selected significantly regulated genes are highlighted.

As potential production‐modulatory genes are expected to show expression differences between high‐ and low‐producing clones, the above‐generated differential expression data were first visualized as volcano plots to identify statistically significant genes. In the non‐induced state, substantially more genes were significantly downregulated than upregulated, with log_2_ FCs calculated as high‐producing versus low‐producing clones (log_2_FC = high/low), such that negative values indicate reduced expression in high producers relative to low producers (Figure 3C). Among the most significantly downregulated genes (Log_2_ FC < −2) were keratin associated protein 19‐1 (KRTAP19‐1), spermidine/spermine N1‐acetyltransferase 1 (SAT1), cellular communication network factor 2 (CCN2), and annexin A1 (ANXA1), whereas inositol polyphosphate‐5‐phosphatase D (INPP5D), Rac family small GTPase 3 (RAC3), and thiol methyltransferase 1A (TMT1A) were strongly significantly upregulated (Log_2_ FC > 2) (Figure 3C). Under induced conditions, the total number of differentially expressed genes was reduced compared with the non‐induced state (Figure 3D). While several genes, including KRTAP19‐1 and SAT1, remained significantly regulated after induction, additional genes such as insulin‐induced gene 1 (INSIG1), protein phosphatase 1 regulatory subunit 15 A (PPP1R15A), nerve growth factor receptor (NGFR), and coiled‐coil domain containing 62 (CCDC62) showed strong FCs. Similarly, beyond RAC3 and TMT1A, RNA polymerase II, I and III subunit L (POLR2L) and myosin VC (MYO5C) were also significantly upregulated following induction (Figure 3D).

Potential engineering targets for modulating LVV production were required to be both differentially expressed and sufficiently abundant. Putative OEX targets were defined as genes upregulated in high‐producing clones (Log_2_FC > 0.6) with TPM ≥ 10. Under non‐induced conditions, 167 genes met these criteria, only two of which showed Log_2_FC > 2.0 (Figure 4A). In induced samples, 252 genes were identified, none exceeding a Log_2_FC of 2.0 (Figure 4A).

**Figure 4:**
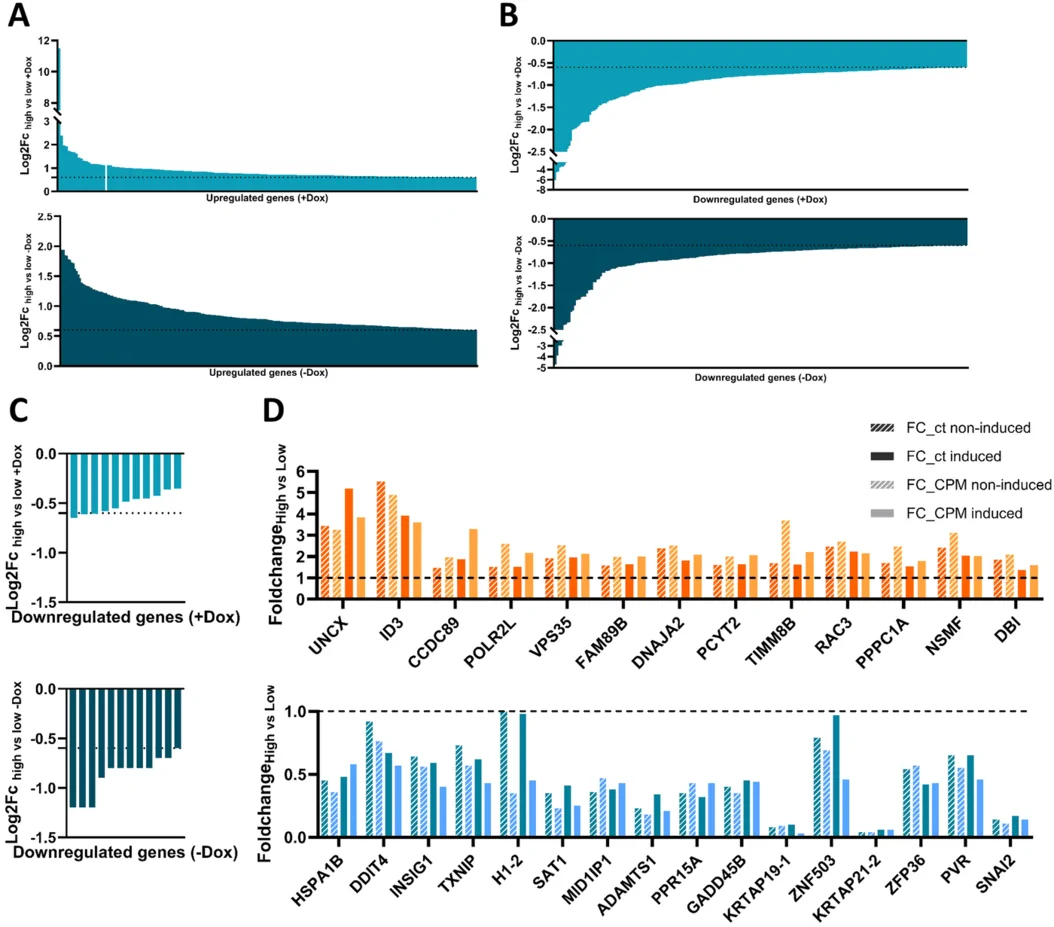
Differential gene expression analysis in response to doxycycline induction. (A–B) Ranked log2 fold changes of upregulated (A) and downregulated (B) genes in induced and non‐induced conditions. (C) Selected downregulated antiviral genes showing consistent expression changes between high‐ and low‐producing clones under both conditions. (D) Comparison of fold changes based on qPCR (CT) and RNA‐seq. (CPM) measurements for selected upregulated (top) and downregulated (bottom) genes under induced and non‐induced conditions.

Putative KO targets were defined as genes downregulated in high‐producing clones (Log_2_FC < −0.6) with TPM ≥ 10. In non‐induced samples, 260 genes fulfilled these criteria, including 12 with strong downregulation (Log_2_FC < −2.0; minimum −6.0) (Figure 4B). Similarly, 237 genes were identified under induced conditions, 11 of which showed Log_2_FC < −2.0 (minimum −4.7) (Figure 4B).

Differential expression of previously described antiviral RFs was also assessed. Under induced conditions, 12 RFs were downregulated in high producers (Log_2_FC < −0.6), including three with pronounced enrichment in low‐producing clones (Log_2_FC < −1.0) (Figure 4C). Under non‐induced conditions, 11 RFs met the same criteria (Figure 4C).

Final engineering targets were selected from genes differentially expressed exclusively under induced conditions or consistently across both states and prioritized based on reported biological function and relevance to LVV production. This resulted in 16 KO, and 13 OEX candidates selected for validation (Tables 4 and 5). In addition, the highly expressed RFs heat shock protein family A (HSPA8), tripartite motif containing 28 (TRIM28), and stearoyl‐CoA desaturase (SCD) were included for functional testing (Table 5).

**Table 4:** List of selected KO targets. Selection criteria are either differential expressed genes (DEG) or restriction factor (RF).

Gene	ENSG number	Log_2_FC (high/low)	TPM (high)	TPM (low)	Selection criteria
HSPA1B	ENSG00000204388	−0.6	1264	2170	DEG
DDIT4	ENSG00000168209	−0.7	831	1454	DEG
INSIG1	ENSG00000186480	−1.2	300	746	DEG
TXNIP	ENSG00000265972	−1.1	187	430	DEG
H1‐2	ENSG00000187837	−1.0	104	232	DEG
SAT1	ENSG00000130066	−1.8	58	229	DEG
MID1IP1	ENSG00000165175	−1.1	98	227	DEG
ADAMTS1	ENSG00000154734	−2.1	41	192	DEG
PPP1R15A	ENSG00000087074	−1.1	81	188	DEG
GADD45B	ENSG00000099860	−1.1	71	163	DEG
KRTAP 19‐1	ENSG00000184351	−4.7	4	117	DEG
ZNF503	ENSG00000165655	−1.0	43	95	DEG
KRTAP 21‐2	ENSG00000187026	−3.9	5	77	DEG
ZFP36L1	ENSG00000185650	−0.8	38	73	DEG
PVR	ENSG00000073008	−1.0	14	31	DEG
SNAI2	ENSG00000019549	−2.6	4	27	DEG
HSP8	ENSG00000109971	0.2	3747	3401	RF
TRIM28	ENSG00000130726	−0.4	900	1227	RF
SCD	ENSG00000099194	−0.2	706	884	RF

**Table 5:** List of selected OEX targets.

Gene	ENSG number	Log_2_FC (high/low)	TPM (high)	TPM (low)
UNCX	ENSG00000164853	1.9	19	5
ID3	ENSG00000117318	1.9	79	24
CCDC89	ENSG00000179071	1.7	23	8
POLR2L	ENSG00000177700	1.1	81	41
VPS35	ENSG00000069329	1.1	64	33
FAM89B	ENSG00000176973	1.0	42	23
DNAJA2	ENSG00000069345	1.1	72	38
PCYT2	ENSG00000069345	1.0	53	28
TIMM8B	ENSG00000150779	1.2	383	190
RAC3	ENSG00000169750	1.1	1133	579
PPP1CA	ENSG00000172531	0.9	99	60
NSMF	ENSG00000165802	1.0	308	112
DBI	ENSG00000155368	0.7	145	99

As the selected engineering targets were identified based on RNA sequencing‐derived expression data, their expression patterns were cross‐validated by qPCR to provide an independent technical validation of selected candidates and to confirm the observed FCs. All OEX candidates showed qPCR FCs greater than 1 in high‐ versus low‐producing clones, closely matching the RNA sequencing–derived FCs (Figure 4D). Likewise, all KO candidates exhibited qPCR FCs below 1, in good agreement with the RNA sequencing data (Figure 4D). Notable exceptions were H1‐2 and ZNF503, which displayed PCR FCs close to or above 1.

Following successful cross‐validation of these carefully selected targets, all gene candidates were taken forward for subsequent engineering of low‐producing clones, with the aim of improving LVV productivity.

### 3.4 Engineering of LVV Production Modulator Genes

To improve LVV yields, the above‐identified target genes were used for OEX or KO in low‐producing clones to assess their potential for enhancing LVV productivity. OEX constructs were successfully generated and validated for four target genes, while for 16 KO targets and the integrated viral HIV gag control, sgRNAs with high cutting efficiency were selected for Cas9‐mediated indel knockout.

To evaluate the potential of the KO target genes to modulate LVV production, sgRNAs/Cas9 RNPs were individually transfected into low‐producing clone 33. Seventy‐two hours post‐transfection, editing efficiencies were assessed by ICE analysis, which deconvolutes Sanger sequencing traces to quantify CRISPR‐induced indels. Most sgRNAs achieved > 50% editing efficiency, ranging from 42% (MID1IP1) to 88% (HIV‐gag) (Figure 5A). Please note that no functional sgRNA was found for KRTAP19‐1, KRTAP21‐2, PVR, and ZFP36L1. These results confirm efficient genome editing across most selected targets and provide a robust basis for subsequent functional evaluation.

**Figure 5:**
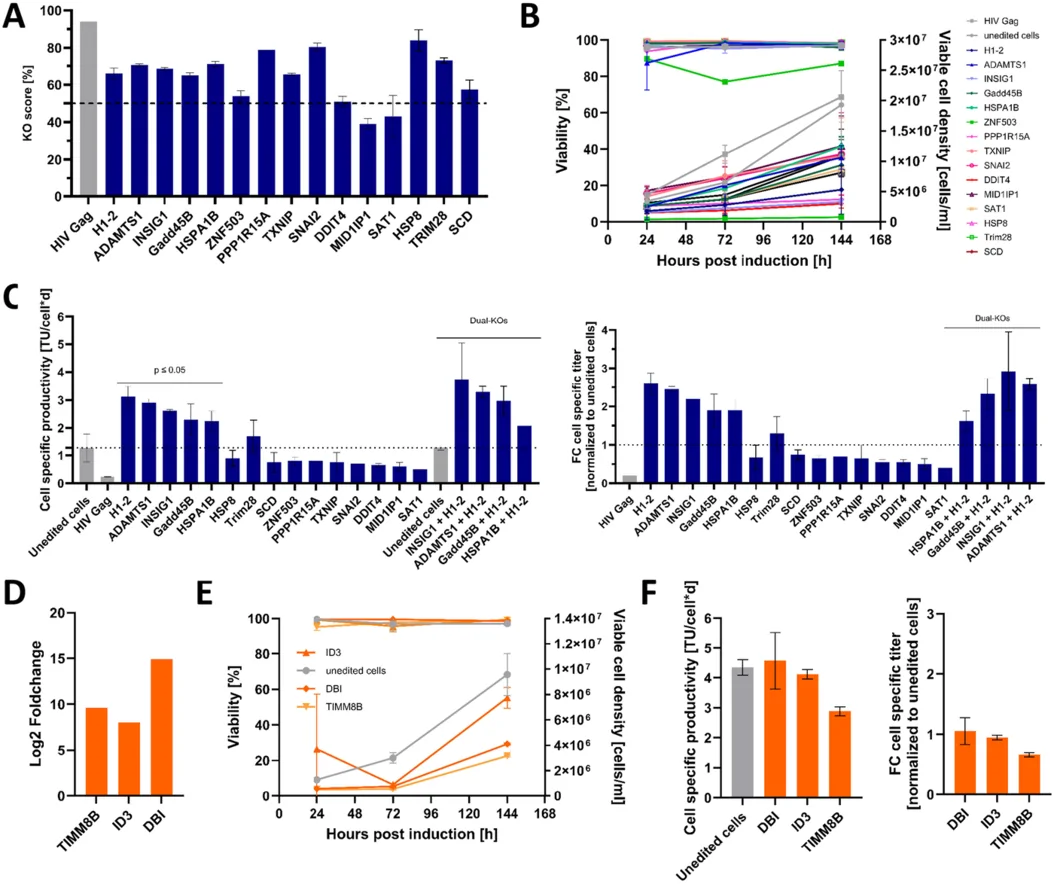
Functional validation of candidate genes affecting productivity. (A) KO efficiency of selected target genes. (B) Cell viability and viable cell density over time following induction. (C) Comparison of cell‐specific productivity (left) and normalized cell‐specific productivity to unedited cells (right) after gene KO. (D) Overexpression of selected OEX targets compared normalized to cells transfected with empty vector (control cells). (E) Time‐course of viability and VCD for selected OEX conditions. (F) Effects of individual gene OEX on cell‐specific productivity (left) and normalized productivity to cells transfected with empty vector (control cells, right).

Seventy‐two hours after CRISPR/Cas9 RNP transfection, editing efficiencies were assessed, and edited cell pools were induced for LVV production. The pools were then cultivated for 6 days to evaluate cell growth, viability, and LVV productivity during the production window. Viability remained above 90% for most conditions, indicating that disruption of the tested genes was generally tolerated during this short‐term production screen. This does not exclude longer‐term selection against edited cells or effects that may become apparent in fully edited clonal lines (Figure 5B). Notable exceptions included ZNF503, which showed a pronounced reduction in viability at 72 h (77%). In contrast, VCD increased over time for most conditions, to varying extents. Control cells (HIV Gag) and KOs such as HSPA1B, GADD45B, and ADAMTS1 showed robust expansion, reaching around 1.1–2.0*10^7^ cells/mL at 144 h, whereas MID1IP1, ZNF503, and TXNIP exhibited reduced growth. H1‐2 and INSIG1 displayed moderate VCD despite high viability, indicating impaired proliferation rather than increased cell death. To account for differences in cell growth, cell‐specific LVV productivity was calculated. KO of H1‐2 resulted in the strongest titer increase (2.6‐fold, 3.1 TU/cell*day), followed by ADAMTS1 (2.5‐fold) and INSIG1 (2.2‐fold) (Figure 5C). Additional increases were detected for GADD45B and HSPA1B (both ~1.9‐fold). In contrast, disruption of the integrated HIV‐gag control significantly reduced productivity, confirming assay specificity.

To assess whether simultaneous disruption of two host–cell factors could result in synergistic improvements in LVV productivity compared to single‐gene knockouts, sgRNAs targeting H1‐2 were co‐transfected with sgRNAs targeting HSPA1B, GADD45B, ADAMTS1, and INSIG1. Editing efficiencies for H1‐2 ranged from 48% to 57%. For the co‐targeted genes, cutting efficiencies were 58% for HSPA1B, 77% for GADD45B, and 62% for INSIG1, whereas ADAMTS1 showed a markedly lower efficiency of 26% in the double knockout setting (Figure S1A). After induction and cultivation, viability remained consistently high (> 90%) and edited cells again exhibited reduced proliferation and lower final VCDs (Figure S1B). LVV productivity was increased for all combinations, with the largest increase observed for INSIG1 + H1‐2 (~ 3.7 TU/cell*day), followed by ADAMTS1 + H1‐2 (2.6‐fold), GADD45B + H1‐2 (2.3‐fold), and HSPA1B + H1‐2 (1.6‐fold) compared with control. The observed increases are consistent with additive or partially overlapping mechanisms.

In parallel, selected candidate genes were evaluated by OEX. Of the initially selected targets, three constructs (DBI, ID3, and TIMM8B) were successfully generated, as other candidates could not be reliably cloned or expressed. OEX was confirmed by qPCR, showing strong upregulation of target transcripts (Figure 5D). Following transient plasmid transfection of clone 33, induction of LVV production, and subsequent cultivation, cell viability remained high across all conditions (Figure 5E). OEX affected cell proliferation more distinctly, with the control condition showing the highest VCD. Among the edited cells, ID3 OEX resulted in the highest VCD, whereas DBI and TIMM8B exhibited a slower increase in VCD over time. Analysis of cell‐specific LVV productivity, however, showed no increase in LVV cell‐specific titer for any target (Figure 5F). OEX of DBI (4.6 TU/cell*day) and ID3 (4.1 TU/cell*day) resulted in productivity levels comparable to the cells transfected with an empty vector (4.3 TU/cell*), while TIMM8B showed reduced productivity. Together, these findings suggest that increasing expression of candidate genes is insufficient to enhance LVV production, whereas removal of inhibitory host‐cell factors represents a more effective engineering strategy.

In summary, the functional screening identified HSPA1B, ADAMTS1, GADD45B, INSIG1, and H1‐2 as promising KO targets for improving LVV production, while OEX of selected candidates did not yield beneficial effects.

## 4 Discussion

LVV producer cell lines represent a scalable platform for gene therapy manufacturing; however, further optimization of stable systems remains necessary to increase vector yields and reduce production demands. In this study, comparative transcriptomic profiling of suspension‐adapted GPRTG cell lines with distinct productivity phenotypes enabled systematic identification of host‐cell determinants associated with LVV output (Klimpel 2024; Throm 2009). Integration of differential gene expression, pathway enrichment, and functional validation revealed key cellular processes distinguishing highly productive from poorly productive clones.

High‐producing clones consistently exhibited upregulation of transcriptional, translational, metabolic, and energy‐associated pathways. These findings are consistent with previous reports demonstrating that efficient viral vector production requires high cellular biosynthetic capacity (Klimpel et al. 2024; Merten et al. 2016; Perry and Rayat 2021).

LVV production imposes a substantial metabolic burden, requiring coordinated activation of RNA synthesis, protein translation, lipid biosynthesis, and ATP generation. Consistent with this, high‐producing clones exhibited increased expression of genes associated with transcriptional, translational, and metabolic pathways compared to low‐producing clones, indicating a biosynthetically active state that supports efficient vector production. In contrast, low‐producing clones showed reduced activation of these pathways, suggesting limited capacity for viral genome expression and particle assembly. In parallel, pathways linked to mitochondrial dysfunction were downregulated in high producers, whereas low‐producing clones displayed signatures indicative of impaired mitochondrial function. Reduced mitochondrial performance in low producers likely limits ATP availability and thereby restricts energetically demanding steps of LVV production.

Notably, productive clones maintained a proliferative phenotype under induced conditions. While LVV production is often associated with growth arrest, our data indicate that sustained proliferation is compatible with efficient vector production. However, in line with previous studies showing increased titers independent of cell growth following multi‐gene KO^17^, proliferation alone does not fully explain productivity differences, highlighting the importance of cellular state rather than cell number (Han et al. 2021).

Transcriptomics‐guided target selection identified genes associated with stress response, lipid metabolism, and transcriptional regulation, including INSIG1, GADD45B, HSPA1B, ADAMTS1, and H1‐2. Functional validation demonstrated that KO of these genes increased cell‐specific LVV productivity by up to 2.6‐fold, establishing a direct link between host‐cell pathway modulation and improved vector output.

These results support the concept that LVV production is constrained by intrinsic host‐cell bottlenecks rather than solely by viral gene expression. In particular, lipid metabolism emerged as a key determinant. INSIG1, a negative regulator of the SREBP–SCAP pathway, controls cholesterol and fatty acid biosynthesis (Zhang et al. 2019). Its disruption likely enhances membrane lipid availability required for viral assembly and budding, consistent with previous findings linking lipid homeostasis to retroviral production. Similarly, chromatin organization represents a critical regulatory layer. Fan et al. and Turner and Margolis reported that a disruption of the linker histone H1‐2 increases transcriptional accessibility of integrated vector elements (Fan et al. 2005; Turner and Margolis 2017). We therefore tested whether H1‐2 disruption increased transcription of integrated viral production cassettes. However, targeted qRT‐PCR analysis of viral components, including gag and tat, did not reveal a consistent increase in transcript abundance after H1‐2 knockout compared with control cells (Figure S2). These results indicate that the enhanced LVV productivity observed after H1‐2 disruption is unlikely to be explained by increased steady‐state transcription of the analyzed viral components. Instead, H1‐2 may influence LVV production through indirect or transcription‐independent mechanisms, such as altered chromatin regulation of host‐cell genes, RNA processing, translation, protein homeostasis, particle assembly, or vector release. Future studies should validate H1‐2 depletion at the protein level and evaluate fully edited clonal producer cell lines to determine the maximal productivity gain and define the underlying mechanism.

In contrast, OEX of selected candidate genes did not improve LVV productivity. While DBI and ID3 maintained production levels comparable to cells transfected with an empty vector, TIMM8B negatively affected growth and productivity. These findings indicate that enhanced expression of productivity‐associated genes is insufficient to improve vector yield, and instead support a model in which removal of inhibitory host‐cell processes is the dominant mechanism driving productivity gains.

The evaluation of double KO combinations further demonstrated that simultaneous targeting of multiple host‐cell pathways can enhance LVV production. The INSIG1 + H1‐2 combination resulted in a 2.9‐fold increase in cell‐specific productivity, slightly exceeding the 2.6‐fold improvement observed for the H1‐2 single KO. This synergistic effect suggests that modulation of complementary processes, such as lipid metabolism and chromatin accessibility, can further relieve cellular constraints on vector production.

However, editing efficiencies of 56%–63% indicate that only a fraction of cells in the population carried both gene disruptions (estimated around 34% for INSIG1 + H1‐2), meaning that the observed productivity likely underestimates the performance of fully edited double KO cells. Increasing editing efficiency may therefore further enhance productivity. Approaches such as sequential transfections, optimized sgRNA design, or clonal selection could increase the fraction of cells carrying multiple gene disruptions and thereby enhance LVV productivity (Doench et al. 2016; Ran et al. 2013).

Consistent with these findings, previous studies have demonstrated stronger synergistic effects using clonal multi‐gene KO cell lines. For example, sequential disruption of OAS1, LDLR, and PKR resulted in up to 7.0‐fold higher LVV titers, whereas individual KOs yielded only around 2.0‐fold improvements (Han et al. 2021). Compared with these approaches, the use of polyclonal pools in the present study likely limits the observed effect size but enables rapid screening of combinatorial targets.

Importantly, the identified host‐cell pathways, including lipid metabolism, stress response, and chromatin organization are not specific to lentiviral systems but are broadly involved in viral replication and particle production (Freed 2015; Goff 2007). This suggests that transcriptomics‐guided host‐cell engineering represents a generally applicable strategy for improving viral vector manufacturing.

In summary, comparative transcriptomic analysis enabled systematic identification of host‐cell processes limiting LVV production. Functional validation demonstrated that targeted disruption of specific host factors significantly enhances cell‐specific productivity. Together, these findings provide a rational framework for host‐cell engineering and support the development of next‐generation LVV producer cell lines through systems‐level optimization.

## Author Contributions

Kerstin Otte, Holger Laux supervised the project. Jona Röscheise planned and conducted experiments. Lena‐Marie Eberle analyzed and evaluated data. Jona Röscheise analyzed and evaluated data and wrote the initial manuscript. All authors read the final manuscript and provided critical feedback.

## Ethics Statement

The experiments of this study were conducted without animal material and, therefore, not critical in regard to ethics.

## Conflicts of Interest

The authors declare no conflicts of interest.

## Supporting information


**Figure S1:** Validation of selected KO genes in combination with H1‐2. (A) KO efficiency of dual KO combinations. (B) Time‐course of viability and VCD following induction for edited and unedited cells.
**Figure S2:** qPCR analysis of viral transcript abundance after H1‐2 knockout. Relative transcript levels of integrated viral components gag and tat were measured by qPCR after H1‐2 KO and normalized to PPIA. Fold changes were calculated relative to control cells using the 2^−ΔΔCp method. Bars show the mean relative expression of two H1‐2 KO samples normalized to the control condition; error bars indicate standard deviation.

## Data Availability

The datasets generated and analyzed during the current study are not publicly available, but are available upon reasonable request from CSL Behring (by contacting Dr. Ellen Otte at ellen.otte@csl.com.au).
